# A 9-year longitudinal study on trajectories of aggressive and depressive symptoms in male and female children with overweight

**DOI:** 10.1186/s13104-019-4734-x

**Published:** 2019-10-30

**Authors:** Luca Cerniglia, Silvia Cimino, Michela Erriu, Stanislav Jezek, Carlos A. Almenara, Renata Tambelli

**Affiliations:** 1 Psychology Faculty, Department of Psychology, International Telematic University Uninettuno, Rome, Italy; 2 Psychology and Medicine Faculty, Department of Dynamic and Clinical Psychology, Sapienza, University of Rome, Rome, Italy; 3 Institute for Research on Children, Youth and Family, Faculty of Social Studies, Masaryk University, Brno, Czech Republic; 4 School of Health Sciences, Universidad Peruana de Ciencias Aplicadas, Chorrillos, Lima, Peru

**Keywords:** Aggressive symptoms, Children, Developmental trajectories, Depressive symptoms, Longitudinal, Overweight

## Abstract

**Objectives:**

The aim of this four waves 9-year longitudinal study was to examine aggressive/depressive symptoms trajectories in a sample of N = 90 children with overweight and a matched group of children with normal weight (subjects balanced by sex and sociodemographic characteristics). Weight and height were measured by pediatricians to calculate body mass index (BMI). Aggressive/depressive symptoms were measured through the Child Behavior Check-List filled out by children’s parents. Multilevel modeling was used to obtain the best fitting curves describing the change over time in aggression and depression scores. These analyses were performed by sex and group.

**Results:**

Children with overweight showed a general increase of aggressive symptoms over time, with a peak at 8 years of age in males, whereas scores of the control group decreased over time both in males and in females. Female children with overweight showed increasing levels of depressive symptoms, with a peak at 8 years of age; children with normal weight, instead, showed low scores at all assessment points. The results highlight the importance of considering the developmental trajectories of aggression and depression in children of different weight status.

## Introduction

Childhood overweight (body mass index—BMI from 85th to less than the 95th percentile) is a major social and health issue [1, 2] that has been indicated as a crucial risk factor for the onset of physical problems (such as cardiovascular diseases, Type II diabetes, asthma, obstructive sleep apnea, and hyperlipidemia), and psychopathological symptoms including internalizing and externalizing difficulties [3, 4].

These evidence point to the importance of monitoring the possible problematic emotional-behavioral functioning associated with childhood overweight [5, 6]. However, only a few longitudinal studies have focused on aggressive/depressive symptoms in male and female children with overweight from early childhood to pre-adolescence [7–9].

Previous research has demonstrated that overweight in children can be associated with several biological and environmental aspects [10]; however, only a few scholars have focused on potential associations between high BMI and psychopathological risk in children over time. Although scarce, this literature suggested that overweight in children can be correlated with depressive and aggressive symptoms [11]. It is noteworthy that those studies that longitudinally addressed this issue typically focused on clinical samples, whereas community samples have been less studied [12, 13]. Previous research has also indicated the usefulness of evaluating possible associations between children’s BMI and psychopathological symptoms, separately for males and females [14].

To respond to these gaps in the literature, this study aimed to examine the developmental trajectories of emotional-behavioral functioning in a sample of children with overweight and normal-weight peers matched for socio-demographic characteristics. In a previous study, we reported the trajectories of these two groups over three waves (2, 5, 8 years of age) and did not include pubertal age [15]. Thus, we could not verify possible variations in the trajectories of weight status in males and females, which have been posited to occur in children around the age of 10/11 years of age, as indicated by Dunger et al. [16]. In line with these considerations, the present contribution adds to the previous, with data from the fourth assessment point (11 years of age) following the same methodology.

## Main text

### Methods: participants and procedure

Following the procedure visible here [10.1371/journal.pone.0190731], children aged 11 (sd = 0.78) were recruited for the fourth wave of the study, thanks to the collaboration of pediatricians working in public and private kindergartens and schools in Central Italy. All subjects were followed up from the previous assessment points and their scores were considered to update the trajectories and aggressive/depressive symptoms considering two groups of children: with normal weight (NW) and with overweight (OW). Pediatricians assessed children’s BMI and children’s parents filled out a measure tapping emotional-behavioral functioning of their offspring (see below). The sample was not affected by attrition thanks to the collaboration of pediatricians and schools, which strongly supported the program.

### Tools

#### BMI index

BMI is a composite measure of children’ weight and height considering age. To identify children with overweight, we used international cut-off points [17].

#### Children’s emotional/behavioral problems

Two versions of the Child Behavior Checklist (CBCL) were used (CBCL 1½–5 and CBCL 6–18 [18, 19]. Both versions contain items assessing internalizing/externalizing problems, although the formulation of some items differs across versions to be coherent with the developmental changes within these domains. Therefore, we chose items from both versions of the CBCL known to have greater developmental invariance and identifying “core” aspects of internalizing and externalizing syndromes that are developmentally appropriate and phenotypically expressed from preschool to puberty [20]. Consequently, we created subsets named “Depression” and “Aggression”, as further described here [15].

### Data analyses

First, to establish grouping criteria for the analyses, we compared the normal-weight with the overweight groups and females with males in outcome measures (i.e., depression and aggression scores at each time point and the average scores across all four-time points). Based on these results, we performed the multilevel modeling analyses to model change over time in depression and aggression levels.

Data analyses were performed using R version 3.5.3 [21]. The *packagenlme* version 3.1-139 [22] was used for multilevel modeling and *ggplot2* version 3.1.1 [23] for data visualization. Exploratory data analyses were performed before multilevel modeling. Model-building was proceeded sequentially with a model contrasting approach, examining the results of model specification, plots (e.g., fitted values versus residuals), the inclusion of covariates and random effects, and considering both parsimony and theoretical sense. ANOVA was used to compare models, and the following criteria were considered to evaluate model fit: Chi square significant test, likelihood ratio test, Akaike information criteria (AIC) value, and the − 2LL (log-likelihood) value. Due to unbalanced data, models were fitted considering residual autocorrelation and heteroscedasticity to select the optimal error structure. The maximum likelihood (ML) method was used to allow ANOVA comparisons between models, whereas the restricted maximum likelihood (REML) method was used as the estimator for the final models.

## Results

Exploratory analyses revealed statistical differences in age (see Additional file 1: Table S1) between the NW group and the OW group at the first time points (*t*(131) = − 2.84, *p* = 0.005, *M*_*dif*_ = − 0.18, 95% CI = − 0.30 to − 0.05), and at the second one (*t*(164) = − 2.99, *p* = 0.003, *M*_*dif*_ = − 0.19, 95% CI = − 0.32 to − 0.06).

Regarding BMI, there were no differences between males and females. In the NW group, males were heavier than females at the first time point (*t*(88) = − 3.12, *p* = 0.002, *M*_*dif*_ = − 1.01, 95% CI = − 1.66 to − 0.37). In the group with overweight, there were not statistical sex differences in BMI. Regarding both aggression and depression scores, the OW group scored higher than the NW group in both measures and in all time points. Males scored higher than females in aggression at the third time point (*t*(133) = − 5.53, *p *< 0.001, *M*_*dif*_= − 4.42, 95% CI = − 5.99 to − 2.84). This pattern was also observed within the OW group (*t*(65) = − 19.80, *p *< 0.001, *M*_*dif*_ = − 9.24, 95% CI = − 10.18 to − 8.31), but not within the NW group. Regarding depression, females scored higher than males in depression at the third time point (*t*(131) = 4.75, *p *< 0.001, *M*_*dif*_ = 2.36, 95% CI = 1.38 to 3.34). This pattern was also observed within the OW group (*t*(75) = 11.36, *p *< 0.001, *M*_*dif*_ = 4.42, 95% CI = 3.65 to 5.20), but not within the NW group.

After these preliminary results, multilevel modeling analyses were performed with the following predictors and covariates: time point, age, sex, and group (i.e., NW and OW), the latter two both as time-invariant characteristics. The time-relevant predictor was time point and group was the main predictor of interest. Aggression and depression were the outcome variables. Aggression and depression scores were skewed (1.2 and 0.97, respectively). To lessen the skew, 0.5 was added to each value and the square root was taken. The resulting skewness was 0.38 for aggression and 0.34 for depression. For both outcome measures, we evaluated several models and ended up with a mixed-effects model with a mixed-effects polynomial (quadratic) regression model with random intercept and random slope, in both cases. The best-fitting models are shown in Table 1.

**Table 1:** Results of the final mixed-effects regression models for aggression and depression

Parameters	Aggression			Depression		
	Estimate	S.E.	*p*	Estimate	S.E.	*p*
Intercept	*2.8*	0.35	< .0001	*2.11*	0.28	< .001
Time	− 0.27	0.26	0.3028	− *0.57*	0.2	0.0053
Time^2	− *0.36*	0.1	0.0003	− 0.003	0.08	0.9632
Age	*0.19*	0.08	0.018	*0.21*	0.06	0.0013
Sex	− 0.46	0.31	0.1433	− 0.19	0.24	0.4361
Group	0.35	0.31	0.2652	0.18	0.24	0.4603
Age*sex	− 0.09	0.08	0.2372	− 0.05	0.06	0.3747
Age*group	0.01	0.08	0.8732	− 0.08	0.06	0.2225
Sex*group	− 0.47	0.43	0.2775	*0.68*	0.33	0.0434
Time^2*age	*0.03*	0.01	0.0013	− 0.004	0.01	0.5721
Time^2*sex	0.18	0.11	0.1034	0.1	0.09	0.2581
Time^2*group	*0.27*	0.11	0.0185	*0.5*	0.09	< .001
Age*sex*group	− 0.12	0.11	0.2795	0.12	0.08	0.1642
Time^2*age*sex	− 0.01	0.01	0.1658	− 0.01	0.01	0.2206
Time^2*age*group	− *0.04*	0.01	0.0009	− *0.07*	0.01	< .001
Time^2*sex*group	*0.56*	0.16	0.0004	− *0.52*	0.12	< .001
Time^2*age*sex*group	− *0.09*	0.02	< .001	*0.08*	0.01	< .001
*Model fit*						
Intercept VAR	0.13			0.14		
Residual VAR	0.26			0.14		
Residual *SD*	0.51			0.38		
AIC	1339.17			1029.52		
BIC	1448.5			1138.85		
− 2LL	1291.17			981.52		

Significant estimates are italic (*p* < 0.05). Aggression and depression scores were transformed to lower skewness. Time^2 is the quadratic term for time. Age was centered on the grand mean. Sex was binary coded: 0 = female, 1 = male. Group was binary coded: 0 = control group, 1 = clinical group. *VAR* variance, *SD* standard deviation, *AIC* Akaike information criteria, *BIC* Bayesian information criterion. − 2LL = negative two log likelihood

**Fig. 1:**
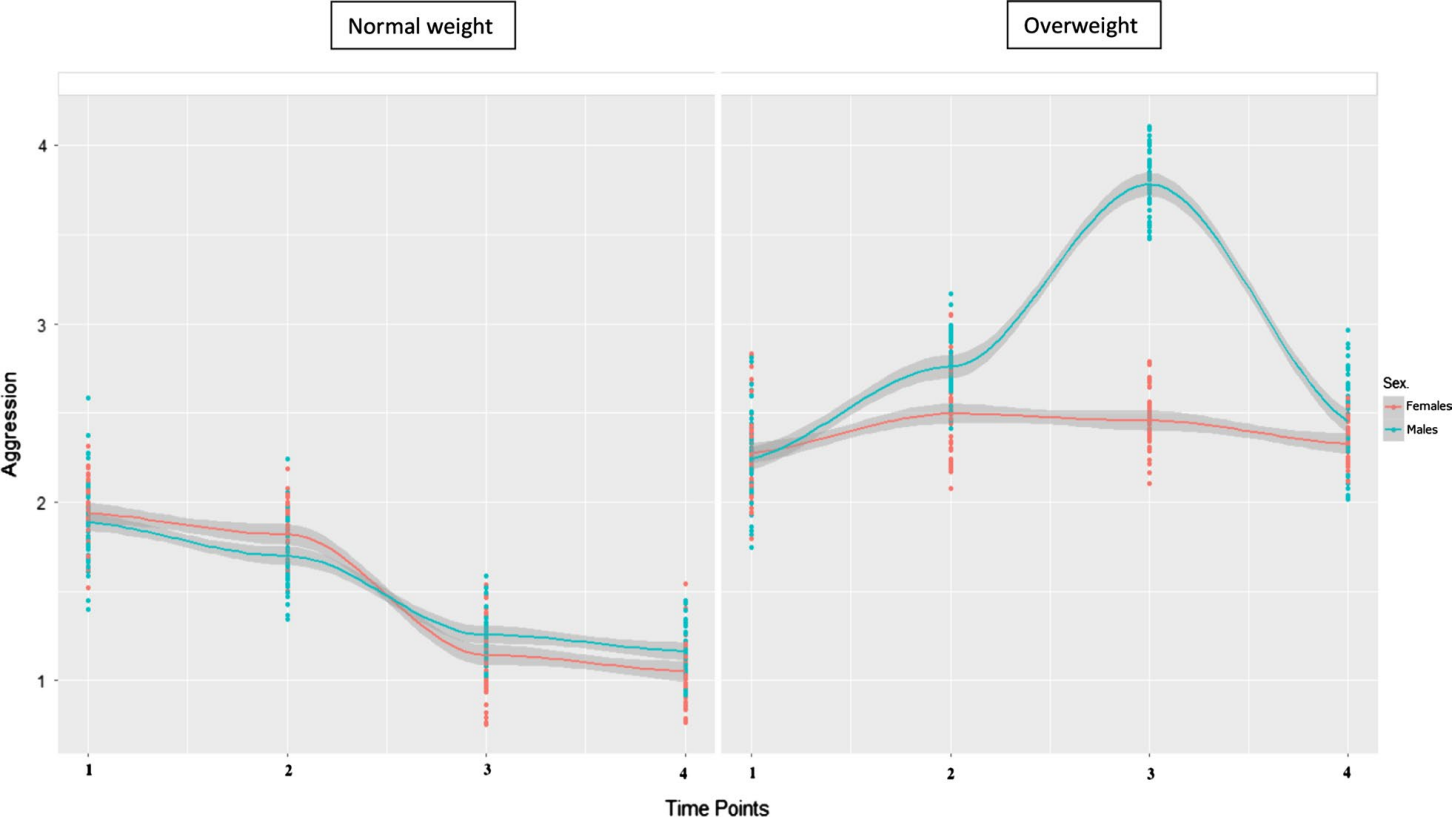
Fitted curves of aggression scores by group and sex across the four time points. Fitted curves include confidence band (grey color). Dispersion of the data (aggression scores), at each time point, is represented by color dots (red for females, blue for males)

**Fig. 2:**
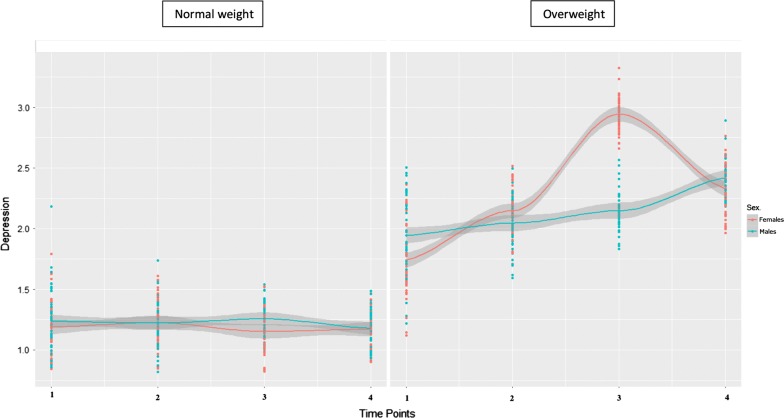
Fitted curves of depression scores by group and sex across the four time points. Fitted curves include confidence band (grey color). Dispersion of the data (depression scores), at each time point, is represented by color dots (red for females, blue for males)

Figures 1 and 2 display the fitted curves obtained from these models.

As can be seen in the Table 1 after controlling for covariates, the largest effect is provided by the non-linear interaction between sex and group over time (+ 0.56 for aggression and − 0.52 for depression), followed by the non-linear effect of group belonging (+ 0.27 for aggression and + 0.50 for depression), time (as a quadratic term, − 0.36 for aggression scores only), and age (0.19 for aggression and 0.21 for depression). In other words, as can be seen in Figs. 1 and 2, the non-linear developmental trajectories of aggression and depression scores in the group with overweight start and end higher than the normal weight group. Moreover, they reveal a peak around age eight in which within-group sex differences are evident in the OW group only. These results complement the descriptive analyses mentioned above in which we found sex differences within the OW group at the third time point in both aggression and depression scores.

## Discussion

The present research note updates the results of a three waves previous longitudinal study [15], by adding a fourth assessment point and contributing to the understanding of aggressive/depressive trajectories in children with overweight and normal weight.

Females with normal weight showed a decreasing trajectory in aggressive symptoms from T1 to T4, as expected from previous literature [24], and it is noteworthy that they showed a larger drop in these scores around 8th years of age. Males showed this pattern too, but their scores at the fourth wave were higher than females. It can be speculated that the specific developmental stage around 8 years of age is associated with protective factors against psychopathological risk, probably due to an increased capacity of emotion regulation, and increased social skills [15]. As for the youths with overweight, their aggressive scores were higher than their NW peers in all the assessment points, with males showing an overall increase over time and females remaining relatively stable. However, aggressive problems in males increased from T1 to T3 and decreased at T4. As above, the third assessment point (8 years of age) seemed to be very significant, with a high peak in maladaptive scores in males, then reducing at 11 years of age. Some authors have underlined that between 6 and 8 years of age physical modifications are particularly significant because children start entering the pre-puberty developmental phase, with important changes in body weight [25] and these changes in growth can be associated with physiological and psychological changes that, if on one hand can pave the way to adapative functioning in normative samples, on the other hand can play a crucial role in the establishment and development of psychopathology in at-risk populations. In the case of 6–8 years old children with overweight, it can be hypothesized that they start to be more aware of the negative attitudes and prejudices that overweight youths may suffer and engage in social comparisons more frequently, with a possible “negative cascade” on their psychological well-being [5]. In children with normal weight, the effect of age seemed to be larger in females, whereas in OW children this effect is very large in males.

A similar pattern was found with regards to depressive symptoms. Children with normal weight showed low and constant levels of depressive problems (almost overlapping in males and females). The subjects with overweight, instead, showed higher scores, with a peak in females’ symptoms around 8 years of age. After this peak, their symptoms followed a decreasing trend. However, females’ scores at T4 were higher than T1.

Two further considerations are noteworthy. First, although aggressive symptoms were overall higher in males (both NW and OW), females’ scores were higher at 2 years of age in this study sample. Similarly, depressive symptoms were overall more problematic in females, but males showed higher scores at T1 and T4, especially in the group with overweight. Second, the specific developmental phase of T3 (8 years of age) seems to have and effect both on the emotional-behavioral functioning of NW and OW group (as described above). However, belonging to the group with overweight seems to magnify this effect, with high peaks in aggressive (in males) and depressive (in females) symptoms at that particular age.

This study has several important implications. Given the above results, pediatricians should consider that children with overweight necessitate attention both from a biological and psychological point of view, being aware the period around 8 years of age seems to be crucial in children’s physical and emotional/behavioral development. This piece of data should be used to inform prevention and intervention programs. Importantly, as noted above, the results of this study showed marked differences in the trajectories of male and female children with overweight, also indicating that the classical view in which males are more aggressive than females and girls are more prone to depressive symptoms [26] does not apply to children with overweight. In line with these results, intervention on psychopathological symptoms in children with overweight should be tailored to children’s sex.

## Limitations

- The study does not allow to define the causal links describing the association between symptoms and overweight.
- Further studies with larger samples are required to increase our knowledge on the influence of weight on children’s emotional-behavioral functioning, particularly with regard to gender differences between 8 and 11 years.

## Supplementary information

**Additional file 1: Table S1.** Descriptive statistics of variables by group and time point.

## Data Availability

The datasets used and analysed during the current study are available from the corresponding author upon reasonable request to the corresponding author.

## Abbreviations

BMI: body mass index
NW: normal weight
OW: overweight
sd: stadard deviation
