# Supplementary material for: A 9-year longitudinal study on trajectories of aggressive and depressive symptoms in male and female children with overweight
Source: BMC Res Notes. 2019 Oct 30;12:710. doi: 10.1186/s13104-019-4734-x (PMC6820918; doi:10.1186/s13104-019-4734-x)
Supplement: Supplementary file 1 — Additional file 1: Table S1. Descriptive statistics of variables by group and time point. [file 13104_2019_4734_MOESM1_ESM.docx]

**Table S1. Descriptive statistics of variables by group and time point**

|  |  |  | Age | | | |  | BMI | | | |  | Aggression | | | |  | Depression | | | |
| --- | --- | --- | --- | --- | --- | --- | --- | --- | --- | --- | --- | --- | --- | --- | --- | --- | --- | --- | --- | --- | --- |
| Time Point | Group |  | *M* | *SD* | Min. | Max. |  | *M* | *SD* | Min. | Max. |  | *M* | *SD* | Min. | Max. |  | *M* | *SD* | Min. | Max. |
| 1 | NW |  | 2.22 | 0.26 | 1.60 | 2.90 |  | 16.08 | 1.61 | 12.62 | 21.34 |  | 3.36 | 2.15 | 0 | 10 |  | 1.09 | 1.14 | 0 | 7 |
|  | OW |  | 2.40 | 0.53 | 1.60 | 3.90 |  | 24.40 | 2.89 | 16.87 | 30.02 |  | 5.16 | 3.15 | 1 | 14 |  | 3.32 | 2.29 | 0 | 8 |
|  |  |  |  |  |  |  |  |  |  |  |  |  |  |  |  |  |  |  |  |  |  |
| 2 | NW |  | 5.10 | 0.49 | 4.10 | 5.90 |  | 16.10 | 0.91 | 14.31 | 18.00 |  | 3.29 | 2.69 | 0 | 12 |  | 1.39 | 1.58 | 0 | 7 |
|  | OW |  | 5.29 | 0.36 | 4.60 | 5.90 |  | 26.01 | 2.38 | 20.19 | 30.06 |  | 6.47 | 3.21 | 1 | 15 |  | 3.99 | 2.05 | 1 | 8 |
|  |  |  |  |  |  |  |  |  |  |  |  |  |  |  |  |  |  |  |  |  |  |
| 3 | NW |  | 7.71 | 0.46 | 7.00 | 8.50 |  | 16.14 | 0.95 | 13.02 | 18.27 |  | 1.04 | 1.17 | 0 | 4 |  | 1.06 | 1.02 | 0 | 3 |
|  | OW |  | 7.80 | 0.42 | 7.00 | 8.60 |  | 25.95 | 1.56 | 22.22 | 29.76 |  | 10.24 | 5.14 | 2 | 16 |  | 6.43 | 2.88 | 2 | 12 |
|  |  |  |  |  |  |  |  |  |  |  |  |  |  |  |  |  |  |  |  |  |  |
| 4 | NW |  | 11.46 | 0.34 | 11.00 | 12.00 |  | 20.93 | 3.66 | 15.04 | 28.84 |  | 0.97 | 1.12 | 0 | 4 |  | 1.06 | 0.96 | 0 | 3 |
|  | OW |  | 11.45 | 0.32 | 11.00 | 12.00 |  | 31.35 | 3.83 | 23.42 | 40.02 |  | 5.47 | 2.86 | 2 | 14 |  | 5.28 | 2.06 | 2 | 10 |

***Note.*** BMI = Body Mass Index.Values for age, aggression and depression are original scores, before their transformation for modeling analisys.
